# Economic Inequality in Outpatient Healthcare Utilization: The Case of Iran

**Published:** 2018-08-20

**Authors:** Sajad Vahedi, Aziz Rezapour, Abolfazl Mohammadbeig, Ardeshir Khosravi

**Affiliations:** ^1^ Department of Health Economics, School of Health Management and Information Sciences, Iran University of Medical Sciences, Tehran, Iran; ^2^ Health Management and Economics Research Center, Iran University of Medical Sciences, Tehran; ^3^ Research Center for Environmental Pollutants, Department of Epidemiology and Biostatistics, Faculty of Health, Qom University of Medical Sciences, Qom, Iran; ^4^ Deputy of Public Health, Ministry of Health and Medical Education, Tehran, Iran

**Keywords:** Healthcare disparities, Health services, Spatial analysis, Iran

## Abstract

**Background:** One of important goals of any health system is to reduce healthcare inequalities in its jurisdiction. We aimed to track economic inequality in outpatient health care utilization after the healthcare transformation plan in Iran.

**Study Design:** A cross-sectional study.

**Methods:** The data obtained from the Iranian healthcare utilization household survey conducted in 2015. The inequality in health care utilization was assessed through concentrating index, concentration curve, and odds ratio. GIS analysis also was used to show provincial concentration index in the map of Iran. The analysis was performed on more than 14000 subjects aged 15 yr or higher reported outpatient health care service’s needs.

**Results:** The richest to poorest odds ratio of outpatient health care utilization was 1.14 (95%CI: 1.11, 1.17). The concentration index of outpatient healthcare utilization was obtained 0.094 (95%CI: 0.77, 0.11). Although the concentration indices of rural and urban residents were significantly different, there was no significant difference between male and female subject. Provincial and GIS analysis showed that inequality in outpatient healthcare utilization was spatially distributed in Iran.

**Conclusions:** Findings of current study indicate that after the healthcare transformation plan, economic inequality in outpatient healthcare utilization still were pro-rich in Iran as a whole and in some of its provinces.

## Introduction

Good health consists of the best attainable average and the smallest feasible differences among individuals and groups^1^. Hence, one of the important goals of any health system is to reduce healthcare inequalities in its jurisdiction ^1,2^. Unfortunately, despite dramatic improvement in access to health care services, there are major disparities in healthcare utilization between different socioeconomic groups^3^. These immoral inequalities not only seen in low-and-middle income countries (LMICs), but also some high-income countries are faced with different degrees of healthcare utilization inequality^4^.

Decrease in health outcomes and increase in health inequity are the true costs of unequal access to healthcare services. This condition could have significant side-effects on the productivity of labor market and decrease gross domestic product (GDP)^5^. Generally, inequality in access to healthcare services could increase both mortality and morbidity^6^. Therefore, universal health coverage (UHC) is extremely recommended as a scheme, regardless of socioeconomic and cultural background, to increase the access to necessary health care services^7^.

Iran is a middle-income country with mixed economy that spends about 7% of its GDP on healthcare8. Previous nationa^14^ and subnational^3^ studies, showed that Iran faced considerable health care utilization inequality. In spite of health equity issues that reflected in the upstream documents^9^ and several healthcare reforms and plans such as family physician program^10^ as well as health sector transformation plan (HTP)^11^, there are the worrying concerns about utilization of necessary health care services by disadvantaged groups. Thus, the purpose of this study was to track inequality in outpatient healthcare services in Iran and among its provinces after HTP by using recent healthcare utilization survey.

## Methods

### Data

Data were obtained from national survey entitled Iranian healthcare utilization household survey (IrUHS) that was conducted by the National Institute Health Research and Statistical Center of Iran in 201512. The IrMIDHS aimed to collect and prepare valid nation-wide data on health and population indices in order to assess the status of health care services and the impact of social indicators on utilization of health care services by Iranians in the health sector of Iran^3^. Multi-stage proportional stratified cluster sampling was used in this survey. However, due to differences in population size of Iranian provinces and their districts, the proportion of each district was determined from total sample size. Subsequently, random samples of clusters in each district were selected and weighted according to the rural and urban population within each region. Each cluster consisted of 10 households^12^.

The IrUHS consist of two questionnaires; household (41 questions) and healthcare utilization. Healthcare utilization questionnaire consisted of two sections that were about utilization of outpatient (38 questions) and inpatient (38 questions) healthcare services by household members. These questionnaires were completed by conducting face-to-face interviews with household members. Overal 22470 household questionnaires and 18984 outpatient section questionnaire were completed. To assess the economic inequalities in health care utilization (outpatient services only) and due to missing data, we excluded people under 15 year. Accordingly, analysis was done for 14785 subjects.

### Measurements of economic Status

The IrUHS have no observation about income or expenditure level; hence we used the wealth index which created through the principal component analysis statistical method. This economic measure has been used successfully in previous studies to measure socioeconomic inequalities and is especially recommended for LMICs^13^. Two categories of variables, including place of resistance (such as home ownership and floor area) and household assets (such as private car, motorcycle, computer, internet use, kitchen, telephone, and central heating machine) used to construct the wealth index. The constructed wealth index was divided into 5 quintiles (poorest, poor, middle, rich, and richest) for using in the subsequent analysis.

### Healthcare utilization measure

As a proxy for health care utilization, we used utilization of outpatient health care services measured in the first section of healthcare utilization questionnaire. Several questions from this survey were used to measure need for healthcare services and utilization of them. In this research, one question from household questionnaire was used as outpatient services’ need (have you needed any outpatient care within two weeks?). On the other hand, one question from healthcare utilization questionnaire was used as utilization of outpatient services (have you used any outpatient services within the last two weeks?)

### Inequality analysis

The concentration index and concentrating curve alongside odds ratio (richest to poorest quintiles odds ratio of healthcare utilization) was used to analyze potential inequality in outpatient healthcare utilization in Iran. Provincial wealth index, concentration index, and odds ratio were also calculated for provinces of Iran. Finally, GIS analysis was used to show provincial concentration index on Iran’s map.

### Concentration curve

The concentration curve was introduced to show how loss or gain in an outcome (healthcare utilization here) is distributed between different economic groups. In this curve, the cumulative percentage of subjects (individuals, households or jurisdictions) is ranked according to their economic status. If the outcome of interest unequally distributed among poor economic groups, the concentration curve will be above the equality line and vice versa. If the outcome equally distributed among studied units, the concentration curve will coincide with line of equality^14^.

### Concentration index

Concentration index is the most famous measure that has been used in the health inequality literature,^15^. This measure could be calculated from the enclosed space between the concentration curve and line of equality. If the concentration curve placed above the line of equality, the index will be negative and vice versa. Equation 1, which is based on Kakvani formula, shows concentration index mathematically.

$$C=2/n.\mu \left[\sum_{i=1}^{n}Y_{i}R_{i}\right]-1$$

In this equation, *Y*_i_ shows the outcome (health care utilization) of the *i*_th_ studied units, *μ* indicates its average, and *R*_i_ represents the fractional rank of the *i*_th_ participants in the economic status distribution. Concentration index varied between -1 and +1, where -1 implies that the studied outcome is entirely concentrated among the poor and vice versa.

The STATA software version 12 was used for statistical analysis. The conindex DO file of STATA software was used to calculate concentration index in aggregate level for Iran and its provinces. This DO file has possibility to compare inequality between levels of modifying factor^16^. GIS analysis was conducted by tmap package of R software.

## Results

Out of 78378 surveyed subjects, 18984 had reported the need for outpatient healthcare services in the last two weeks. Needs of 10222 (66.4%) subjects, aged 15 yr or older have been met. Majority of studied units were female (59.57%) and lived in urban areas (66.16%) (Table 1).

**Table 1:** Summary statistics for healthcare utilization (outpatient healthcare services) based on data from the Iranian healthcare utilization survey 2015

**Variables**	**Number**	**Percent**
Healthcare utilization		
Yes	10222	69.1
No	4563	30.9
Sex		
Male	5978	40.4
Female	8807	59.6
Area of residence		
Rural	5003	33.8
Urban	9782	66.2
Wealth index		
Poorest	2,965	20.3
Poor	3,057	20.6
Middle	2,977	20.1
Rich	2,836	19.2
Richest	2,956	2.0

The rates of outpatient utilization of healthcare services are shown across the wealth quintiles. Subjects with higher economic status had higher rate of outpatient healthcare utilization. Moreover rates of healthcare utilization regarding to sex and residence area of subjects were studied. The rate of healthcare utilization in males, females, and rural as well as urban areas was higher in the rich quintiles (Table 2).

**Table 2:** Estimated concentration index, odds ratio and their standard error of health care utilization with regard to the subgroup analysis in Iran, 2015

**Variables**	**Wealth quintiles**					**Richest to poorest Quintiles**		**Concentration** **index**		**Concentration** **indices**	
	**Poorest**	**Poor**	**Middle**	**Rich**	**Richest**	**Odds Ratio (95% CI)**	**p value**	**Odds Ratio (95% CI)**	**p value**	**Diff.**	**P value**
Healthcare utilization (SD)	0.62 (0.48)	0.66 (0.47)	0.68 (0.46)	0.70 (0.45)	0.74 (0.43)	1.14 (1.11, 1.17)	0.001	0.09 (0.77, 0.11)	0.001		
Healthcare utilization- sex (SD)	Male	0.58 (0.49)	0.63 (0.48)	0.661 (0.47)	0.65 (0.48)	0.74 (0.43)	1.17 (1.12, 1.22)	0.001	0.11 (0.08, 0.14)	0.001	-0.032	0.072
	Female	0.64 (0.48)	0.68 (0.46)	0.70 (0.45)	0.72 (0.44)	0.74 (0.43)	1.12 (1.08, 1.16)	0.001	0.08 (0.06, 0.10)	0.001		
Healthcare utilization- living area (SD)	Urban	0.64 (0.43)	0.68 (0.45)	0.70 (0.45)	0.70 (0.45)	0.75 (0.43)	1.12 (1.08, 1.16)	0.001	0.08 (0.06, 0.10)	0.001	-0.039	0.039
	Rural	0.60 (0.48)	0.63 (0.48)	0.65 (0.47)	0.66 (0.47)	0.67 (0.48)	1.07 (1.02, 1.13)	0.003	0.04 (0.01, 0.07)	0.006		

**Figure 1:**
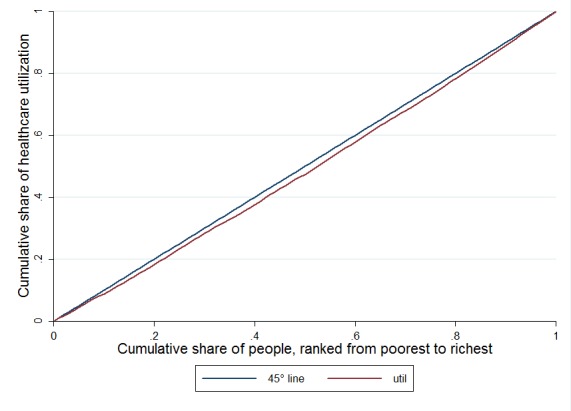
Concentration curve for healthcare utilization in Iran, 2015

The concentration index and odds ratio for outpatient healthcare utilization and regarding sex and living area are also summarized in Table 2. The concentration index for healthcare utilization was obtained as 0.094 (95%CI: 0.77, 0.11). This statistically significant concentration index implies that outpatient healthcare utilization concentrated among subjects with higher economic status. In addition the odds ratio of outpatient utilization indicated that subjects with higher economic status have more chance to use these healthcare services. The corresponding concentration curve of healthcare utilization is also depicted in Figure 1.

The concentration index regarding male, female, and urban as well as rural area was calculated as 0.115, 0.083, 0.082 and 0.042, respectively. All of these concentration indices were statistically significant. According to showed indices, the size of these pro-rich inequalities in outpatient healthcare utilization was higher in males and the people living in the urban areas. The difference between concentration indices also reported in Table 2. Only differences between rural and urban concentration indices were statistically significant. The result of odds ratio for sex and area of residence also showed that the subjects with higher wealth quintiles have larger chance to use outpatient health care services.

**Figure 2:**
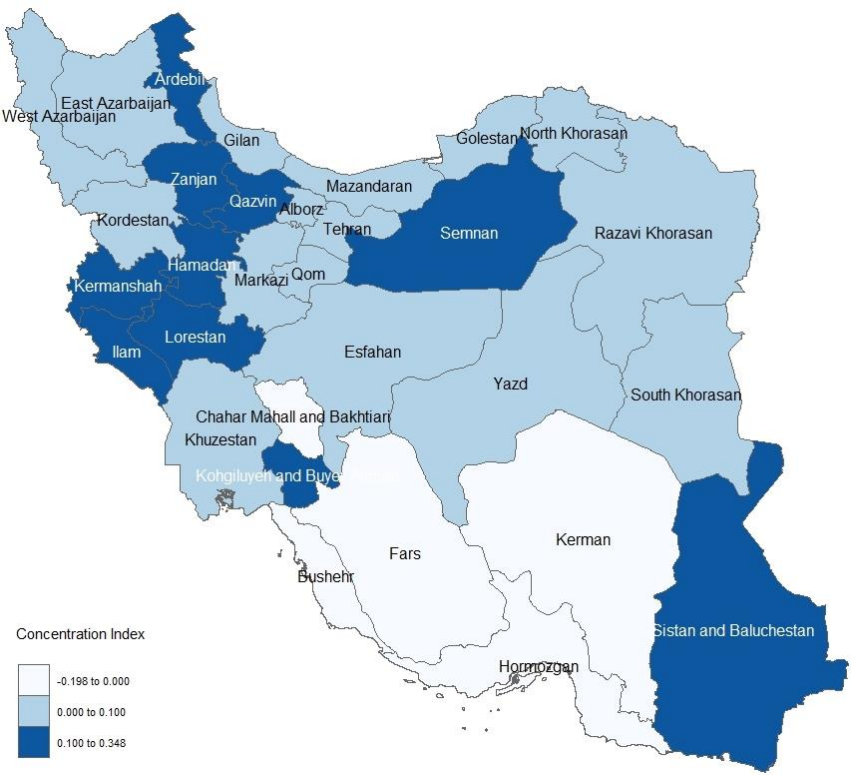
Provincial concentration index of healthcare utilization

Table 3 shows the outpatient healthcare utilization rate, its standard error for different economic quintiles, and measures of economic inequality in health care utilization for each province. The relative difference in outpatient healthcare utilization rates between highest and lowest quintiles was statistically significant in provinces such as Markazi, Mazandaran, Kermanshah, Khozestan, Lorestan, Kordestan, Razavi Khorasan, Ilam, Zanjan, Chaharmahal and Bakhtiari, Golestan, Ardabil, and Qazvin. Among the provinces with a statistically significant odds ratio, the ratio varied from 0.84 in Chaharmahal and Bakhtiari to 1.73 in Semnan. Based on the concentration index, except for Markazi and Kordestan, inequality in outpatient health care utilization was also statistically significant in above mentioned provinces and Hamadan. This measure in these provinces ranged from 0.348 in Kohgiloye and Boyerahmad to -0.138 in Chaharmahal and Bakhtiari. The corresponding provincial concentration index of healthcare utilization was illustrated in the map of Iran in the Figure 2.

**Table 3:** Estimated healthcare utilization in economic quintiles, odds ratio and concentration index by province, Iran 2015

**Province**	**Health care utilization (SD), wealth quintiles**					**Richest to poorest** **quintiles odds ratio**		**Concentration index**	
	**Poorest**	**Poor**	**Middle**	**Rich**	**Richest**	**Value** **(95%CI)**	**P value**	**Value** **(95%CI)**	**P value**
Markazi	0.77 (0.42)	0.80 (0.38)	0.82 (0.38)	0.78 (0.41)	0.93 (0.40)	1.26 (1.01, 1.57)	0.034	0.08 (-0.01, 0.18)	0.072
Gilan	0.72 (0.45)	0.71 (0.45)	0.79 (0.40)	0.73 (0.44)	0.81 (0.39)	1.12 (0.76, 1.04)	0.129	0.07 (-0.02, 0.17)	0.112
Mazandaran	0.50 (0.50)	0.50 (0.50)	0.52 (0.50)	0.51 (0.50)	0.64 (0.48)	1.16 (1.03,1.30)	0.009	0.09 (0.00, 0.18)	0.031
East Azarbaijan	0.69 (0.46)	0.62 (0.48)	0.75 (0.43)	0.60 (0.49)	0.76 (0.42)	1.10 (0.97, 1.25)	0.132	0.08 (-0.00, 0.17)	0.055
West Azarbaijan	0.52 (0.50)	0.57 (0.49)	0.67 (0.47)	0. 75 (0.43)	0.79 (0.40)	1.03 (0.91, 1.64)	0.612	0.02 (-0.07, 0.11)	0.656
Kermanshah	0.58 (0.49)	0.69 (0.46)	0.62 (0.48)	0.60 (0.49)	0.64 (0.48)	1.41 (0.91, 1.64)	0.000	0.22 (0.131, 0.31)	0.000
Khozestan	0.70 (0.45)	0.64 (0.47)	0.61 (0.48)	0.67 (0.47)	0.57 (0.49)	1.11 (1.21,1.64)	0.021	0.09 (0.02, 0.16)	0.009
Fars	0.77 (0.41)	0.77 (0.41)	0.74 (0.44)	0.78 (0.41)	0.71 (0.45)	0.94 (0.83, 1.06)	0.357	-0.04 (-0.12, 0.03)	0.244
Kerman	0.61 (0.48)	0.67 (0.46)	0.57 (0.49)	0.50 (0.50)	0.55 (0.49)	0.90 (0.93,1.18)	0.044	-0.07 (-0.15, 0.01)	0.080
Razavi Khorasan	0.61 (0.48)	0.70 (0.45)	0.66 (0.47)	0.73 (0.44)	0.75 (0.42)	1.15 (1.01,1.30)	0.031	0.08 (0.00, 0.17)	0.029
Esfahan	0.75 (0.43)	0.73 (0.44)	0.67 (0.47)	0.68 (0.46)	0.80 (0.39)	1.05 (0.93,1.18)	0.379	0.03 (-0.04, 0.10)	0.356
Sistan and Balochestan	0.54 (0.49)	0.61 (0.49)	0.84 (0.36)	0.64 (0.48)	0.56 (0.50)	1.18 (1.02,1.38)	0.026	0.152 (0.04, 0.260)	0.052
Kordestan	0.73 (0.44)	0.66 (0.47)	0.71 (0.45)	0.92 (0.27)	0.84 (0.37)	1.32 (1.04,1.68)	0.019	0.098 (-0.029, 0.22)	0.124
Hamadan	0.57 (0.49)	0.56 (0.49)	0.70 (0.45)	0.70 (0.46)	0.63 (0.48)	1.15 (0.97,1.36)	0.102	0.11 (0.00, 0.22)	0.042
Chaharmahal and Bakhtiari	0.77 (0.41)	0.80 (0.40)	0.63 (0.48)	0.63 (0.48)	0.67 (0.47)	0.84 (1.03,1.4)	0.024	-0.13 (-0.24, 0.03)	0.008
Lorestan	0.59 (0.49)	0.63 (0.48)	0.70 (0.45)	0.69 (0.46)	0.79 (0.41)	1.23 (1.07, 1.40)	0.002	0.16 (0.07, 0.26)	0.000
Ilam	0.36 (0.48)	0.35 (0.47)	0.51 (0.50)	0.46 (0.50)	0.67 (0.47)	1.39 (1.14,1.71)	0.001	0.17 (0.04, 0.30)	0.007
Kohgiloye and boyerahmad	0.44 (0.49)	0.60 (0.49)	0.71 (0.45)	0.70 (0.45)	0.80 (0.39)	1.47 (1.26, 1.73)	0.000	0.348 (0.23, 0.45)	0.000
Boshehr	0.86 (0.34)	0.89 (0.30)	0.82 (0.38)	0.78 (0.41)	0.80 (0.39)	0.86 (0.69, 1.06)	0.172	-0.073 (-0.17, 0.02)	0.132
Zanjan	0.69 (0.46)	0.79 (.041)	0.88 (0.31)	0.92 (0.27)	0.96 (0.17)	1.82 (1.42,2.33)	0.000	0.24 (0.15, 0.33)	0.000
Semnan	0.42 (0.50)	0.90 (0.29)	0.88 (0.32)	0.82 (0.38)	0.91 (0.28)	1.73 (1.16,2.56)	0.006	0.22 (0.09, 0.35)	0.000
Yazd	0.74 (0.44)	0.74 (0.44)	0.90 (0.29)	0.88 (0.31)	0.82 (0.38)	1.17 (0.89, 1.55)	0.250	0.06 (-0.04, 0.16)	0.230
Hormozgan	0.70 (0.45)	0.62 (0.48)	0.63 (0.48)	0.56 (0.49)	0.59 (0.49)	0.87 (0.76,1.00)	0.052	-0.12 (-0.23, -0.01)	0.026
Tehran	0.82 (0.38)	0.73 (0.44)	0.86 (0.34)	0.82 (0.38)	0.82 (0.37)	1.06 (0.91, 1.25)	0.399	0.04 (-0.02, 0.11)	0.219
Ardabil	0.51 (0.50)	0.71 (0.46)	0.73 (0.44)	0.69 (0.46)	0.78 (0.41)	1.26 (1.06, 1.49)	0.006	0.14 (0.04, 0.23)	0.004
Qom	0.54 (0.50)	0.71 (0.45)	0.62 (0.48)	0.58 (0.49)	0.63 (0.48)	1.01 (0.79, 1.13)	0.862	0.04 (-0.08, 0.16)	0.498
Qazvin	0.39 (0.49)	0.51 (0.50)	0.65 (0.47)	0.58 (0.49)	0.69 (0.46)	1.29 (1.12, 1.49)	0.000	0.168 (0.06, 0.26)	0.008
Golestan	0.46 (0.50)	0.50 (0.50)	0.56 (0.49)	0.49 (0.50)	0.59 (0.49)	1.11 (1.00, 1.24)	0.047	0.088 (0.00, 0.17)	0.045
North Khorasan	0.74 (0.44)	0.83 (0.37)	0.90 (0.30)	0.78 (0.41)	0.81 (0.39)	1.05 (0.78, 1.41)	0.719	0.032 (-0.09, 0.16)	0.615
South Khorasan	0.57 (0.49)	0.75 (0.43)	0.60 (0.49)	0.69 (0.46)	0.70 (0.46)	1.07 (0.90,1.28)	0.398	0.062 (-0.06, 0.18)	0.331
Alborz	0.72 (0.45)	0.76 (0.42)	0.67 (0.47)	0.80 (0.39)	0.75 (0.43)	1.05 (0.89, 1.25)	0.519	0.028 (-0.06, 0.12)	0.561

## Discussion

Based on our knowledge, this research is the first to investigate inequality in outpatient health care utilization simultaneously in Iran and across its provinces. There is a direct relationship between utilization of outpatient services and economic status in Iran and within some of its provinces.

In the aggregate level, utilization of outpatient services is concentrated among the rich subjects. According to another measure of inequality, people with higher economic status have greater chance to benefit from these services. This finding is in line with previous studies that conducted in Iran^3,4^ and other nations^17-19^. Compared with other studies in Iran,^4^, it seems that after HTP^11^, the outpatient healthcare utilization inequality in Iran still concentrated among the rich households. This indicates that this policy has no desirable effect on health care inequality. This study is not in line with former study^20^ that conducted in the west of Iran that showed the inequality in public healthcare utilization was pro-poor in Kermanshah.

In this research, males and females with higher economic status had greater chance to use required health care services. The results of concentration index also confirmed this finding. This is in accordance with former researches that conducted in Iran,^4^. Male subjects have greater concentration index. This seems logical; females have higher healthcare needs^21^ and may have higher rate of healthcare utilization. There are no significant differences between concentration indices of male and females. This highlighted that Iran has no gender disparity in the utilization of outpatient health care services. Place of residence is another factor that may have affects healthcare inequalities^22^. In this study, utilization of outpatient services was concentrated among rural and urban rich quintiles. Urban residents have greater concentration index that was statistically significant. This seems logical; urban households in Iran have higher income inequality and this may worsen health care inequalities such as inequality in utilization of required healthcare services.

Healthcare inequality could be understood through spatial analysis. The findings of this research showed that outpatient healthcare utilization inequality was spatially distributed in Iran. Subjects that ranked in higher economic status in provinces such as Mazandaran, Kermanshah, Razavi Khorasan, Khozestan, Lorestan, Ilam, Semnan, Zanjan, Kohgiloye and Boyerahmad, Kordestan, Ardabil, and Qazvin, had greatest odds to utilize from required health care services. The results of concentration index also confirmed that utilization of outpatient services were concentrated among rich residents of these provinces. This means that in the local level, HTP could not eliminate pro-rich inequality of outpatient healthcare services. National and subnational policy maker must pay attention to this issue and not only provide and finance more human and physical health resources in these provinces but also increase regional policy making to boost health equity. On the other hand, both inequality measures showed that outpatient healthcare inequality in Chaharmahal and Bakhtiari, and Hormozgan was pro-poor. This may be due to immigration of people with higher socioeconomic status to benefit from healthcare services in neighboring provinces.

The findings of this study revealed that after of the HTP Iranians still suffer from pro-rich inequality of outpatient services. This experience highlighted that only spending money could not guarantee health sector reforms. Different social, political and economic factors may have different impacts on effectiveness of UHC programs^23^. Hence, it is strongly recommended that intersectoral action^24^ must be increased in Iran to correct HTP in future.

This study has some limitation that must be acknowledged. It is clear that by using direct economic variables such as income or consumption level of households, inequality analysis could be able to show potential inequalities more effectively. However, in this research the asset index approach, which widely used in previous studies from developing nations, was used to rank studied households. Therefore, it is recommended that future survey in Iran include both monetary and asset index questions. Considering that this study has cross-sectional design, interpretations should be made with caution.

## Conclusion

Economic inequality in healthcare utilization was pro-rich in Iran as a whole and in some of its provinces. On the other hand, two provinces simultaneously had pro-rich healthcare inequality. It seems that the healthcare inequality has spatial pattern in Iran. Examining why this inequality favors the better-off in these provinces deserves special attention. Intersectoral cooperation also recommended correcting HTP in future.

## Acknowledgements

This study was part of a Ph.D. thesis supported by the Iran University of Medical Sciences (grant no. IUMS/SHMIS_20169321504002). The authors hereby would like to thank Mr. Muhammad Shamsaldini and Dr. Nahid Aghaei for their valuable guidance. The authors also thank the Iran National Institute of Health Research (NIHR) for their support and for free access to the original data of UHS.

## Conflict of interest statement

None of the authors has any financial or other interests that might influence the conduct of the study or accurate reporting of the results.

## Funding

No commercial or sponsor support was used for current study.

## Highlights

- One of important goals of any health system is to reduce healthcare inequalities in its jurisdiction.
- After Healthcare Transformation Plan, Iranians still suffer from pro-rich outpatient healthcare utilization inequality.
- It seems that the healthcare inequality has spatial pattern in Iran.
- Intersectoral action also recommended correcting HTP in future.

## References

[1] World Health Organization. The world health report 2000: health systems: improving performance: World Health Organization; 2000.

[2] Rezapour A, Ebadifard Azar F, Azami Aghdash S, Tanoomand A, Hosseini Shokouh SM, Yousefzadeh N (2015). Measuring equity in household's health care payments (Tehran-Iran 2013): technical points for health policy decision makers. Med J Islam Repub Iran.

[3] Mohammadbeigi A, Hassanzadeh J, Eshrati B, Rezaianzadeh A (2013). Socioeconomic inequity in health care utilization, Iran. J Epidemiol Glob Health.

[4] Hajizadeh M, Connelly LB, Butler James RG, Khosravi A (2012). Unmet need and met unneed in health care utilisation in Iran. Int J Soc Econ.

[5] Mackenbach JP, Meerding WJ, Kunst AE (2011). Economic costs of health inequalities in the European Union. J Epidemiol Community Health.

[6] Korda RJ, Butler JR, Clements MS, Kunitz SJ (2007). Differential impacts of health care in Australia: trend analysis of socioeconomic inequalities in avoidable mortality. Int J Epidemiol.

[7] Boerma T, Eozenou P, Evans D, Evans T, Kieny MP, Wagstaff A (2014). Monitoring progress towards universal health coverage at country and global levels. PLoS Med.

[8] Rezaei S, Fallah R, Kazemi Karyani A, Daroudi R, Zandiyan H, Hajizadeh M (2016). Determinants of healthcare expenditures in Iran: evidence from a time series analysis. Med J Islam Repub Iran.

[9] Vosoogh Moghaddam A, Damari B, Alikhani S, Salarianzedeh M, Rostamigooran N, Delavari A (2013). Health in the 5th 5-years Development Plan of Iran: Main Challenges, General Policies and Strategies. Iran J Public Health.

[10] Khayatzadeh-Mahani A, Takian A (2014). Family physician program in Iran: considerations for adapting the policy in urban settings. Arch Iran Med.

[11] Mahdavi M, Parsaeian M, Jaafaripooyan E, Ghaffari S (2017). Recent Iranian Health System Reform: An Operational Perspective to Improve Health Services Quality. Int J Health Policy Manag.

[12] Ali Akbari Saba R, Safakish M, Safakish Z, Khabiri Nemati R, Zahedian A, Khosravi A, et al. Utilization of Health Services (UHS) 2015. Tehran: Ministry of Health and Medical Education 2016. [in Persian].

[13] Vyas S, Kumaranayake L (2006). Constructing socio-economic status indices: how to use principal components analysis. Health Policy Plan.

[14] Wagstaff A, Paci P, van Doorslaer E (1991). On the measurement of inequalities in health. Soc Sci Med.

[15] Ramezani Doroh V, Vahedi S, Arefnezhad M, Kavosi Z, Mohammadbeigi A (2015). Decomposition of Health Inequality Determinants in Shiraz, South-west Iran. J Res Health Sci.

[16] O'Donnell O, O'Neill S, Van Ourti T, Walsh B (2016). conindex: Estimation of concentration indices. Stata J.

[17] van Doorslaer E, Wagstaff A, van der Burg H, Christiansen T, De Graeve D, Duchesne I (2000). Equity in the delivery of health care in Europe and the US. J Health Econ.

[18] van Doorslaer E, Koolman X, Jones AM (2004). Explaining income-related inequalities in doctor utilisation in Europe. Health Econ.

[19] Dorjdagva J, Batbaatar E, Dorjsuren B, Kauhanen J (2015). Income-related inequalities in health care utilization in Mongolia, 2007/2008-2012. Int J Equity Health.

[20] Rezaeian S, Hajizadeh M, Rezaei S, Ahmadi S, Kazemi Karyani A, Salimi Y (2018). Measuring and Explaining Socioeconomic Inequalities in Public Healthcare Utilization in Western Iran: Evidence from a Cross-sectional Survey. J Res Health Sci.

[21] Doyal L (2001). Sex, gender, and health: the need for a new approach. BMJ.

[22] Lahana E, Pappa E, Niakas D (2011). Do place of residence and ethnicity affect health services utilization? evidence from greece. Int J Equity Health.

[23] Borgonovi E, Compagni A (2013). Sustaining Universal Health Coverage: The Interaction of Social, Political, and Economic Sustainability. Value Health.

[24] Ndumbe-Eyoh S, Moffatt H (2013). Intersectoral action for health equity: a rapid systematic review. BMC Public Health.

